# Comparison of Allogeneic Stem Cell Transplant and Autologous Stem Cell Transplant in Refractory or Relapsed Peripheral T-Cell Lymphoma: A Systematic Review and Meta-analysis

**DOI:** 10.1001/jamanetworkopen.2021.9807

**Published:** 2021-05-27

**Authors:** Jun Du, Dandan Yu, Xinle Han, Lijun Zhu, Zoufang Huang

**Affiliations:** 1 State Key Laboratory of Experimental Hematology, Institute of Hematology and Blood Disease Hospital, Chinese Academy of Medical Sciences and Peking Union Medical College, Tianjin, People’s Republic of China; 2 Shenzhen PKU-HKUST Medical Center, Shenzhen, People’s Republic of China; 3 Binjiang College of Nanjing University of Information Science & Technology, Jiangsu, People’s Republic of China; 4 Department of Hematology, The First Affiliated Hospital of Gannan Medical University, Jiangxi, People’s Republic of China

## Abstract

**Importance:**

Hematopoietic stem cell transplant (HSCT) is an advisable option for refractory or relapsed peripheral T-cell lymphoma (R/R-PTCL), but whether allogeneic HSCT or autologous HSCT is more beneficial is unknown.

**Objective:**

To compare the effectiveness and safety of allogeneic HSCT vs autologous HSCT in patients with R/R-PTCL.

**Data Sources:**

A systematic search of the PubMed, Embase, the Cochrane Central Register of Controlled Trials, Wanfang, and China National Knowledge Infrastructure databases with the search items *refractory or relapsed peripheral T-cell lymphoma*, *ASCT/autologous stem-cell transplantation*, *allo-HSCT/allogeneic stem-cell transplantation*, *therapeutic effect*, and *treatment* was conducted for articles published from January 12, 2001, to October 1, 2020.

**Study Selection:**

After duplicate and irrelevant publications were discarded, 329 were ineligible according to the inclusion (clinical trials or retrospective studies with >10 samples) and exclusion criteria (articles without overall survival [OS], progression-free survival [PFS], and transplantation-related mortality [TRM]). Thirty trials were included in the meta-analysis. The study followed the Preferred Reporting Items for Systematic Reviews and Meta-analyses (PRISMA) reporting guideline.

**Data Extraction and Synthesis:**

Data on study design, individual characteristics, and outcomes were extracted. All statistics were pooled by applying a random-effects model.

**Main Outcomes and Measures:**

The prespecified main outcomes were OS, PFS, and TRM.

**Results:**

Of 6548 articles, data extracted from the 30 studies (including 880 patients who underwent allogeneic HSCT and 885 who underwent autologous HSCT) were included in this meta-analysis. In the allogeneic HSCT group, a 3-year OS of 50% (95% CI, 41%-60%) and PFS of 42% (95% CI, 35%-51%), a 5-year OS of 54% (95% CI, 47%-62%) and PFS of 48% (95% CI, 40%-56%), and a 3-year TRM of 32% (95% CI, 27%-37%) were observed. In the autologous HSCT group, a 3-year OS of 55% (95% CI, 48%-64%) and PFS of 41% (95% CI, 33%-51%), a 5-year OS of 53% (95% CI, 44%-64%) and PFS of 40% (95% CI, 24%-58%), and a 3-year TRM of 7% (95% CI, 2%-23%) were observed.

**Conclusions and Relevance:**

In this systematic review and meta-analysis, OS and PFS were similar in the allogeneic HSCT and autologous HSCT groups; however, allogeneic HSCT was associated with specific survival benefits among patients with R/R-PTCL.

## Key Points

**Question:**

Is allogeneic hematopoietic stem cell transplant (HSCT) or autologous HSCT more effective and safer for patients with refractory or relapsed peripheral T-cell lymphoma?

**Findings:**

In this systematic review and meta-analysis of 30 trials with 1765 patients, for patients undergoing allogeneic HSCT, the 3-year overall survival was 50%, the 3-year progression-free survival was 42%, and the 3-year transplant-related mortality was 32%. For patients undergoing autologous HSCT, the 3-year overall survival was 55%, the 3-year progression-free survival was 41%, and the 3-year transplant-related mortality was 7%.

**Meaning:**

These findings suggest that allogeneic HSCT may have better effectiveness but be less safe than autologous HSCT for patients with refractory or relapsed peripheral T-cell lymphoma.

## Teaser

This meta-analysis compares the effectiveness and safety of allogeneic vs autologous hematopoietic stem cell transplant in patients with refractory or relapsed peripheral T-cell lymphoma.

## Introduction

Peripheral T-cell lymphomas (PTCLs), a rare and heterogeneous group of non-Hodgkin lymphomas, have a dismal prognosis.^1^ The main subtypes are PTCL not otherwise specified, angioimmunoblastic T-cell lymphoma, anaplastic large-cell lymphoma, and natural killer/T-cell lymphoma and are typically treated with conventional regimens for aggressive B-cell lymphomas, resulting in poor clinical outcomes.^2^ Furthermore, frequent relapses and initially refractory diseases are not uncommon in PTCL, making it more challenging. A study^3^ reported poor survival outcomes for 153 patients with refractory or relapsed PTCL (R/R-PTCL) receiving chemotherapy without hematopoietic transplantation, with a median overall survival (OS) of 13.7 months and progression-free survival (PFS) of 5 months. So far, because of the multitudinous morphologic features of the subtypes and the lack of randomized clinical trials, treatment of this disease remains a challenge, especially for R/R-PTCL.^4^

Given the extremely poor results of current treatments, many researchers have pursued exploratory strategies. Although molecular targeted drugs, such as brentuximab vedotin, provide objective hope for CD30^+^ PTCL, hematopoietic stem cell transplant (HSCT) continues to be a reasonable option. Currently, the concept of high-dose chemotherapy followed by autologous HSCT during first remission in patients with PTCL has been widely accepted by clinical practitioners.^5,6,7^ However, the roles of autologous HSCT and allogeneic HSCT in R/R-PTCL remain far more controversial. Although HSCT has certain beneficial effects, adverse events (AEs) will negatively influence survival as well.^8^ Therefore, this meta-analysis was performed to compare the efficacy and safety of autologous HSCT vs allogeneic HSCT in R/R-PTCL.

## Methods

### Search Strategy and Study Selection

**Figure 1.**
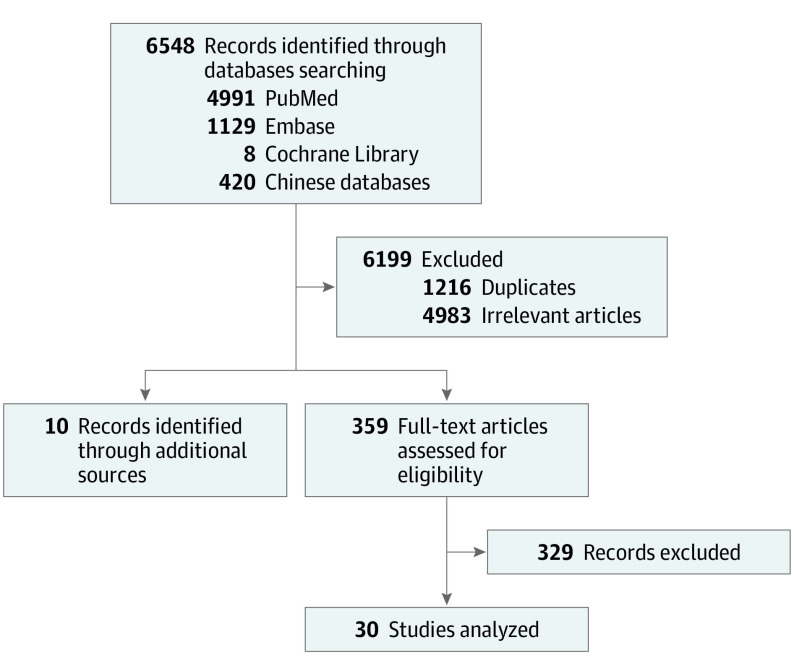
Search Strategy

Our search strategy is shown in in Figure 1. We searched the Cochrane Central Register of Controlled Trials, Embase, PubMed, Wanfang, and China National Knowledge Infrastructure databases with the search terms *refractory or relapsed peripheral T*-*cell lymphoma/refractory or relapsed lymphoma*, *peripheral T-cell/refractory or relapsed T-cell lymphoma*, *peripheral R/R-PTCL*, *ASCT/autologous stem-cell transplantation, allo-HSCT/allogeneic stem-cell transplantation, therapeutic effect/effectiveness/efficacy*, and *treatment*. All records from January 12, 2001, to October 1, 2020, were included. We quantitatively controlled for the factors listed above as well as others we deemed relevant. No language restrictions were applied on retrieval. The protocol was registered with the International Prospective Register of Systematic Reviews (PROSPERO). The study was performed in accordance with the Declaration of Helsinki,^9^ with prior approval of the institutional review board and the ethics committee of each hospital. This study followed the Preferred Reporting Items for Systematic Reviews and Meta-analyses (PRISMA) reporting guideline.

### Inclusion Criteria

All studies were independently evaluated by 2 authors (D.Y. and X.H.). Subsequently, we inspected and discussed the outcomes to reach an agreement. We selected studies based on the following criteria: (1) clinical trials (including randomized clinical trials and nonrandomized clinical trials) and retrospective analytic studies with more than 10 samples; (2) patients with R/R-PTCL (no sex or age restrictions); (3) patients treated with autologous HSCT or allogeneic HSCT; and (4) providing outcome measurements, such as complete response (CR), partial response (PR), OS, PFS, and transplantation-related mortality (TRM). Prospective studies are clinical trials that were all registered online. Retrospective studies include a review of medical records from registry centers.

### Data Extraction

We extracted the following information: (1) basic research information, including the study type, journal, author, areas, year of publication, and number of cases; (2) main characteristics, including age, histologic findings, prior chemotherapy, and complications; and (3) main outcome measurements, including CR, PR, overall response rate, OS, PFS (including event-free survival [EFS] and disease-free survival [DFS]), duration of response, TRM, nonrelapse mortality (NRM), and graft-vs-host disease (GVHD). Two researchers (D.Y. and X.H.) extracted the data independently.

### Statistical Analysis

We performed a meta-analysis for each outcome using the meta package in R software, version 3.6 (R Foundation for Statistical Computing). Because most of the eligible studies we enrolled in were single-arm tests, we calculated single ratios and integrated ratios with 95% CIs. Furthermore, meta-analysis requires that the distribution type of a single rate should be a normal distribution. If not, it must be transformed to follow or be close to a normal distribution to improve the reliability of the combined results. Therefore, we transformed the data we extracted via R software by 5 methods, which were without conversion, logarithmic conversion, logit conversion, arcsine conversion, and Freeman-Tukey double arcsine conversion. After comparing the *P* values of the Shapiro-Wilk test calculated over the data transformed by each method, we chose the most suitable one to determine the integrated results. Considering there were inevitable limitations in the study, such as the diverse populations, we choose the random-effects model as our default regardless of the Cochran *Q* test results.

### Quality Assessment and Bias Risk

We evaluated the quality of all evidence using the Methodological Index for Non-Randomized Studies (MINORS) scale (eFigure 1 in the Supplement) and plotted a heat map using OriginPro software, version 2020b (OriginLab Corp). According to the MINORS scale, 8 methodologic items were estimated for noncomparative studies, and 4 other criteria were estimated for comparative studies. The items are scored 0 (not reported), 1 (reported but inadequate), or 2 (reported and adequate); 16 and 24 are the global ideal scores for nonrandomized and randomized studies, respectively.^10^ We evaluated publication bias by assessing the symmetry of the funnel plot and by performing the Begg test and the Egger test, and we conducted sensitivity analysis as well with R software, version 3.6.

## Results

A total of 6548 studies describing autologous HSCT or allogeneic HSCT for R/R-PTCL were included, including 10 other relevant references identified from additional sources. As shown, 1216 duplicates were discarded, and 5332 remaining articles underwent title and abstract screening to remove irrelevant records. A total of 359 full-text studies were available for us to assess after excluding 4983 unrelated articles. A total of 329 publications did not fulfill the inclusion and exclusion criteria; therefore, our final analysis included 30 trials (7 prospective and 23 retrospective) (Figure 1). However, only 2 trials were comparative studies that evaluated the efficiency and safety of autologous HSCT vs allogeneic HSCT treatment for R/R-PTCL. Therefore, the autologous HSCT group and allogeneic HSCT groups have 16 publications each. Our study included 1765 patients in total; 885 patients underwent autologous HSCT treatment, and 880 patients underwent allogeneic HSCT treatment.

We used a bubble chart (eFigure 2A and B in the Supplement) to show the numbers of patients over the years. Basically, the patient number of the allogeneic HSCT group showed wavelike increases with each passing year. For the autologous HSCT group, there was no apparent association between the patient number and the years. We provide an overview of the literature in Table 1 and Table 2, including basic characteristics. In the autologous HSCT group, the age ranged from 4 to 73 years, and the follow-up time ranged from 1 to 278 months. For the allogeneic HSCT group, the age ranged from 16 to 74 years, and the follow-up time ranged from 0.3 to 195 months. All patients enrolled were no older than 75 years when they received HSCT.

**Table 1.** Overview of the Literature on Allogeneic HSCT in R/R-PTCL

Source	Study type/country	No. of patients/age, median (range), y	Follow-up (range), mo	Histologic subtype	Status before HSCT	Regimen (MA/RIC)	Response	OS (95% CI)	DFS (including EFS and PFS) (95% CI)	TRM/NRM	GVHD
Mamez et al,^11^ 2020	Retrospective study/Switzerland	285/50 (16-69)	72 (69-80)	PTCL-NOS 39%; AITL 29%; ALCL 15%; other 17%	CR 62%; PR 27%	RIC 62%; MA 38%	NA	59% (53%-65%) at 4 y	PFS 54% (48%-61%) at 4 y	TRM 28% at 4 y	aGVHD II-IV 30%; cGVHD 37%; eGVHD 15%
McIlroy et al,^12^ 2020	Retrospective study/United Kingdom	21/45 (24-72)	95	PTCL-NOS 35%; AITL 20%; ALCL 25%; other 20%	CR/PR 90%; PD 10%	RIC 76%; MA 24%	NA	42% at 5 y	NA	NRM, 10% at 1 y	aGVHD 24%; cGVHD 33%
Zhenyang,^13^ 2019	Retrospective study/China	21/37 (12-51)	47 (14-105)	PTCL-NOS 38%; AITL 14%; ALCL 14%; NK/TCL 24%; other 10%	PR 43%; SD/PD 57%	MA 100%	CR 38%; PR/PD 38%	47% (25%-66%) at 3 y	PFS 46% (24%-66%) at 3 y	NRM 24% at 3 y	aGVHD II-IV 14%; cGVHD 19%; eGVHD 14%
Wulf et al,^14^ 2019	Retrospective study/Germany	84/50 (17-74)	15 (2-114)	PTCL-NOS 36%; AITL 20%; ALCL 18%; NK/TCL 5%; other 21%	CR 15%; PR 42%; PD/refractory 26%; SD 17%	MA 100%	CR 54%; PR 8%; PD 20%	38% (33%-44%) at 3 y	DFS 37% (32%-43%) at 3 y	NRM 32% at 3 y 46% at 5 y	NA
Wang et al,^15^ 2019	Retrospective study/China	23/35 (17-59)	29 (0.3-102)	PTCL-NOS 17%; AITL 22%; ALCL 13%; NK/TCL 22%; other 26%	Refractory/relapse 100%	TBI-cyclophosphamide/ busulfan- cyclophosphamide; carmustine, etoposide, cytarabine, and melphalan/ fludarabine and busulfan	CR 30%; PR 22%; SD 9%; PD 39%	43% (30%-69%) at 3 y	PFS 39% (24%-65%) at 3 y	TRM 57%	aGVHD I-III 48%; cGVHD 67%; eGVHD 17%
Huang et al,^16^ 2017	Retrospective study/China	24/37 (16-52)	26	PTCL-NOS 71%; AITL 4%; ALCL 4%; NK/TCL 21%	CR 8%; PR 25%; NR 67%	TBI- cyclophosphamide/ busulfan- cyclophosphamide	CR 58%; PR 29%; NR 67%	53% at 3 y	PFS 49% at 3 y	NRM 18% at 1 y	aGVHD 38%; cGVHD 17%
Corradini et al,^17^ 2014	Prospective study/Italy	23/48 (24-61)	44	PTCL-NOS 54%; AITL 23%; ALCL 20%; other 3%	CR 87%; PR 13%	RIC 100%	CR 70%;	69% at 4 y	PFS 69% at 4 y	TRM 13% at 4 y	aGVHD II-IV 40%; cGVHD 52%; eGVHD 9%
Smith et al,^18^ 2013	Retrospective study/US	126/38 (15-60)	49 (3-157)	PTCL-NOS 50%; AITL 10%; ALCL 40%	CR 30%; relapse 31%	RIC 36%; MA 59%	CR 14%	46% at 3 y	PFS 37% at 3 y	NRM 34% at 3 y	NA
Czajczynska et al,^19^ 2013	Retrospective study/Germany	24/54 (11-65)	42	PTCL-NOS 38%; AITL 21%; ALCL 17%; other 24%	CR 38%; PR 42%; refractory 4%; relapse 12%	RIC 100%	CR 83%; PR 8%	42% (25%-71%) at 3 y	NA	TRM 25%	aGVHD II-IV 25%; cGVHD 30%; eGVHD 12%
Dodero et al,^20^ 2012	Retrospective study/Italy	52/47 (15-64)	67 (18-138)	PTCL-NOS 45%; AITL 17%; ALCL 21%; other 17%	CR/PR 75%; refractory 25%	RIC 100%	NA	50% (36%-63%) at 5 y	PFS 40% (27%-53%) at 5 y	NRM 12%, at 5 y	aGVHD II-IV 22%; cGVHD 23%; eGVHD 6%
Zain et al,^21^ 2011	Retrospective study/US	24/40 (7-72)	49 (16-100)	PTCL-NOS 33%; AITL 17%; ALCL 25%; other 25%	CR 25%; PR 21%; relapse 21%; refractory 33%	RIC 58%; MA 42%	NA	60% at 5 y	PFS 50% (38%-60%) at 5 y	NRM 17%	aGVHD I-IV 50%; cGVHD 67%; eGVHD 54%
Jacobsen et al,^22^ 2011	Prospective study/US	52/46 (24-72)	49 (20-157)	PTCL-NOS 38%; AITL 10%; ALCL 12%; other 40%	CR 44%; PR 31%; relapse 10%; refractory 15%	RIC 40%; MA 60%	NA	41% (28%-55%) at 3 y	PFS 30% (18%-43%) at 3 y	NRM 27% at 3 y	aGVHD II-IV 21%; cGVHD 37%; eGVHD 27%
Shustov et al,^23^ 2010	Prospective study/Italy	17/57 (18-73)	40 (4-96)	PTCL-NOS 41%; AITL 24%; ALCL 6%; other 29%	Refractory/relapse 82%	RIC 100%	CR 71%; PD 24%	59% at 3 y	PFS 53% at 3 y	NRM 19% at 3 y	aGVHD II-IV 24%; cGVHD 41%
Le Gouill et al,^24^ 2008	Retrospective study/France	77/36 (12-61)	43 (4-195)	PTCL-NOS 35%; AITL 14%; ALCL 35%; other 16	CR 40%; PR 30%;	MA 74%	CR 40%; PR 34%	57% (45%-68%) at 5 y	EFS 53% (41%-64%) at 5 y	TRM 34% at 5 y	aGVHD III/IV 21%
Wulf et al,^25^ 2005	Retrospective study/Germany	10/45 (23-53)	7 (4-16)	PTCL-NOS 40%; AITL 20%; ALCL 30%; other 10%	PD/refractory 100%	RIC 100%	CR 70%; PR 10%; SD 10%	70% at 7 mo	PFS 60% at 7 mo	TRM 30% at 7 mo	aGVHD I-III 50%; cGVHD 50%
Corradini et al,^26^ 2004	Prospective study/Italy	17/41 (23-60)	28 (3-57)	PTCL-NOS 53%; AITL 24%; ALCL 23%	CR/PR 82%; PD 12%; relapse 6%	RIC 100%	CR 71%; PR 6%; SD 6%	81% (62%-100%) at 3 y	PFS 64% (39%-89%) at 3 y	NRM 6% at 2 y	aGVHD I-IV 36%; cGVHD 41%; eGVHD 6%

Abbreviations: aGVHD, acute graft-vs-host disease; AITL, angioimmunoblastic T-cell lymphoma; ALCL, anaplastic large cell lymphoma; cGVHD, chronic graft-vs-host disease; CR, complete remission; DFS, disease-free survival; EFS, event-free survival; eGVHD, extensive GVHD; HSCT, hematopoietic stem cell transplant; MA, myeloablative conditioning; NK/TCL, NK/T-cell lymphoma; OS, overall survival; PD, progressive disease; PFS, progression-free survival; PR, partial remission; PTCL, peripheral T-cell lymphoma; PTCL-NOS, peripheral T-cell lymphoma not otherwise specified; RIC, reduced intensity conditioning; R/R-PTCL, refractory or relapsed peripheral T-cell lymphoma; SD, stable disease; TBI, total body irradiation; TRM/NRM, transplantation-related mortality/nonrelapse morality.

**Table 2.** Overview of the Literature on ASCT in R/R-PTCL

Source	Study type/country	No. of patients/age, median (range), y	Median, follow-up, mo	Histologic subtype	Status before HSCT	Regimen (MA/RIC)	Response	OS	DFS (including EFS and PFS)	TRM/NRM
Domingo-Domènech et al,^5^ 2020	Retrospective study/Europe	65/44 (20-71)	35 (3-71)	ALCL 100%	Relapse/refractory 100%	Carmustine, etoposide, cytarabine, and melphalan	NA	83% (74%-92%) at 1 y; 73% (63%-86%) at 3 y	PFS 71% (60%-83%) at 1 y; 64% (52%-78%) at 3 y	NRM 2% at 3 y
Yamasaki et al,^27^ 2019	Retrospective study/Japan	112/57 (18-70)	37 (1-127)	PTCL-NOS 100%	Relapse 100%	MCEC/LEED/ranimustine, etoposide, cytarabine, and melphalan	NA	72% (59%-81%) at 1 y; 49% (35%-61%) at 3 y	PFS 43% (30%-54%) at 1 y; 28% (18%-40%) at 3 y	NRM 5% at 1 y
Roerden et al,^28^ 2019	Retrospective study/Germany	18/59 (21-71)	163 (48-278)	PTCL-NOS 22%; ALCL 33%; AITL 33%; other 12%	Relapse 100%	Carmustine, etoposide, cytarabine, and melphalan	NA	77% at 5 y	PFS 61% at 5 y	TRM 75% at 5 y
Huang et al,^16^ 2017	Retrospective study/China	43/40 (7-63)	31	PTCL-NOS 47%; ALCL 42%; NK/T 12%	CR 60%; PR 17%; NR 23%	MAG/carmustine, etoposide, cytarabine, and melphalan	CR 65%; PR 21%; NR 9%	20% at 3 y	PFS 20% at 3 y	NRM 7% at 1 y
Wang et al,^29^ 2016	Retrospective study/China	32/31 (12-58)	31(1-96)	PTCL-NOS 41%; ALCL 41%; AITL 18%	CR 77%; PR/NR 23%	MA	CR 90%; NR 10%	62% at 5 y	PFS 61% at 5 y	TRM 42% at 5 y
Smith et al,^18^ 2013	Retrospective study/America	115/43 (4-60)	71 (3-167)	PTCL-NOS 34%; ALCL 53%; AITL 13%	CR 56%; relapse 27%	BEAM/TBI based	CR 35%	59% (49%-68%) at 3 y	PFS 47% (37%-56%) at 3 y	NRM 6% at 3 y
d'Amore et al,^30^ 2012	Prospective study/Northern Europe	115/57 (22-67)	60 (26-96)	PTCL-NOS 39%; ALCL 19%; AITL 19%; other 23%	CR 63%; PR 37%	Carmustine, etoposide, cytarabine, and melphalan/carmustine, etoposide, cytarabine, and cyclophosphamide	CR 78%; PR 8%	56% (48%-63%) at 3 y; 51% (43%-59%) at 5 y	PFS 48% (40%-56%) at 3 y; 44% (36%-52%) at 5 y	TRM 4%
Nickelsen et al,^31^ 2009	Prospective study/Germany	33/48 (20-60)	53	PTCL-NOS 33%; ALCL 39%; AITL 12%; other 16%	CR 49%; PR 6%	MegaCHOEP	CR 49%; PR 6%; SD 9%; PD 27%	45% (27%-63%) at 3 y	EFS 26% (10%-41%) at 3 y	TRM 6%
Reimer et al,^32^ 2009	Prospective study/Germany	55/46 (30-65)	33 (5-58)	PTCL-NOS 39%; ALCL 16%; AITL 33%; other 12%	CR 73%; PR 27%	TBI-cyclophosphamide/CHOP	CR 87%; PR 13%	71% at 3 y	DFS 53% at 3 y	TRM 4%
Chen et al,^33^ 2008	Retrospective study/US	10/38 (25-73)	59 (12-138)	PTCL-NOS 40%; ALCL 30%; AITL 10%; NK/T 20%	Refractory 100%	CHOP/DHAP/ESHAP	NA	30% at 5 y	PFS 0% at 5 y	NA
Smith et al,^34^ 2007	Retrospective study/US	32/44 (16-69)	30 (8-95)	PTCL-NOS 34%; ALCL 66%	CR/PR 19%; refractory 26%; relapse 55%	Busulfan, cyclophosphamide, and etoposide	NA	34% at 5 y	NA	TRM 19% at 3 mo
Kim et al,^35^ 2007	Retrospective study/South Korea	40/44 (18-68)	16 (5-135)	PTCL-NOS 50%; ALCL 13%; AITL 7%; NK/T 25%; other 5%	CR 28%; PR 62%; refractory 10%	Carmustine, etoposide, cytarabine, and cyclophosphamide/carmustine, etoposide, cytarabine, and melphalan/busulfan, cyclophosphamide, and etoposide;/etoposide and cyclophosphamide	CR 60%; PR 10%; PD 20%; SD 5%	46% at 1 y	NA	NA
Kewalramani et al,^36^ 2006	Retrospective study/US	24/48 (24-73)	72	PTCL-NOS 58%; ALCL 17%; AITL 17%; other 8%	Relapse 67%; refractory 33%	TBI based 50%; chemotherapy only 50%	CR 63%; PR 37%	33% at 5 y	PFS 24% at 5 y	NA
Kevin^37^ 2003	Retrospective study/Canada	36/46 (19-62)	42 (6-116)	PTCL-NOS 55%; ALCL 25%; AITL 6%; other 14%	Relapse 81%; refractory 19%	TBI-cyclophosphamide/melphalan and etoposide	CR 42%; PR 50%; <PR 8%	48% (31%-66%) at 3 y	EFS 37% (20%-53%) at 3 y	TRM 17%
Rodríguez et al,^38^ 2003	Retrospective study/Spain	115/41 (13-72)	37 (1-133)	PTCL-NOS 63%; ALCL 22%; AITL 5%; other 10%	CR 32%; relapse/refractory 68%	Carmustine, etoposide, cytarabine, and cyclophosphamide/carmustine, etoposide, cytarabine, and melphalan/TBI- cyclophosphamide/cyclophosphamide, etoposide, and carmustine	CR 86%; PR 5%; SD 3%; PD 6%	56% (45%-67%) at 5 y	DFS 60% (49%-71%) at 5 y	TRM 18% at 3 y
Blystad et al,^39^ 2001	Retrospective study/Sweden	40/42 (16-61)	36 (7-100)	PTCL-NOS 50%; ALCL 35%; AITL 5%; other 10%	CR 70%; PR 30%	Carmustine, etoposide, cytarabine, and cyclophosphamide/carmustine, etoposide, cytarabine, and melphalan/TBI-cyclophosphamide	CR 80%	58% at 3 y	EFS 48% at 3 y	TRM 8%

Abbreviations: AITL, angioimmunoblastic T-cell lymphoma; ALCL, anaplastic large cell lymphoma; ASCT, autologous stem cell transplant; CHOP, cyclophosphamide, vincristine, doxorubicin, and prednisone; CR, complete remission; DFS, disease-free survival; DHAP, dexamethasone, cytarabine, and cisplatin; EFS, event-free survival; ESHAP, etoposide, methylprednisolone, cytarabine, and cisplatin; HSCT, hematopoietic stem cell transplantation; LEED, melphalan, cyclophosphamide, etoposide, and dexamethasone; MA, myeloablative conditioning; MAG, cytarabine, mitoxantrone, and recombinant human granulocyte colony-stimulating factor; MCEC, ranimustine, carboplatin, etoposide, and cyclophosphamide; MegaCHOEP, cyclophosphamide, adriamycin, vincristine, etoposide, and prednisone; OS, overall survival; PD, progressive disease; PFS, progression-free survival; PR, partial remission; PTCL, peripheral T-cell lymphoma; PTCL-NOS, peripheral T-cell lymphoma not otherwise specified; RIC, reduced intensity conditioning; R/R-PTCL, refractory or relapsed peripheral T-cell lymphoma; SD, stable disease; TBI, total body irradiation; TRM/NRM, treatment-related mortality/nonrelapse morality.

We used the MINORS scale to assess study quality, and the results are shown in eFigure 3 in the Supplement. The final scores for each study ranged from 11 to 14. Overall, the studies included in the meta-analysis were of relatively good reliability. Four funnel plots (eFigure 4 in the Supplement) were used to estimate all articles’ publication bias. We drew 4 plots for 4 groups, into which all studies were divided based on the survival outcomes concerning OS and PFS at 3 or 5 years. Two small studies with extreme distributions exist, making them asymmetrical. However, we still selected those studies. On the one hand, funnel plots are usually used for comparative studies to identify bias, whereas studies included in our article are mostly single-arm trials; however, those studies that yielded asymmetry could be selected. On the other hand, because HSCT is an exploratory approach for patients with R/R-PTCL and the studies in this field are few, we included all studies, which might have affected the results. We conducted a sensitivity analysis for the 2 comparative studies, which reported opposite findings, and the results are shown in the eFigure 5 in the Supplement.

**Figure 2.**
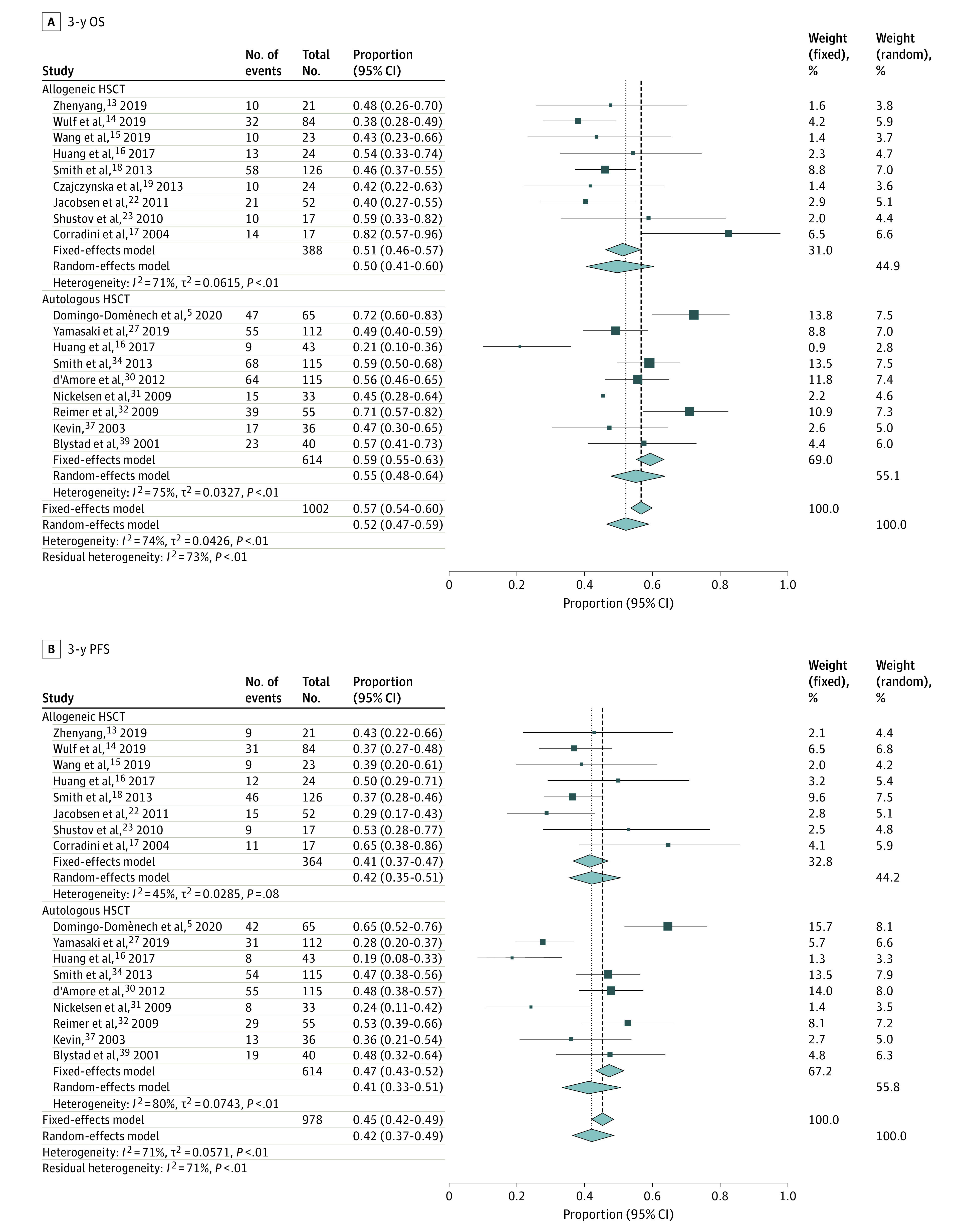
Three-Year Survival Outcomes in Patients with Refractory or Relapsed Peripheral T-Cell Lymphoma HSCT indicates hematopoietic stem cell transplant; OS, overall survival; PFS, progression-free survival.

**Figure 3.**
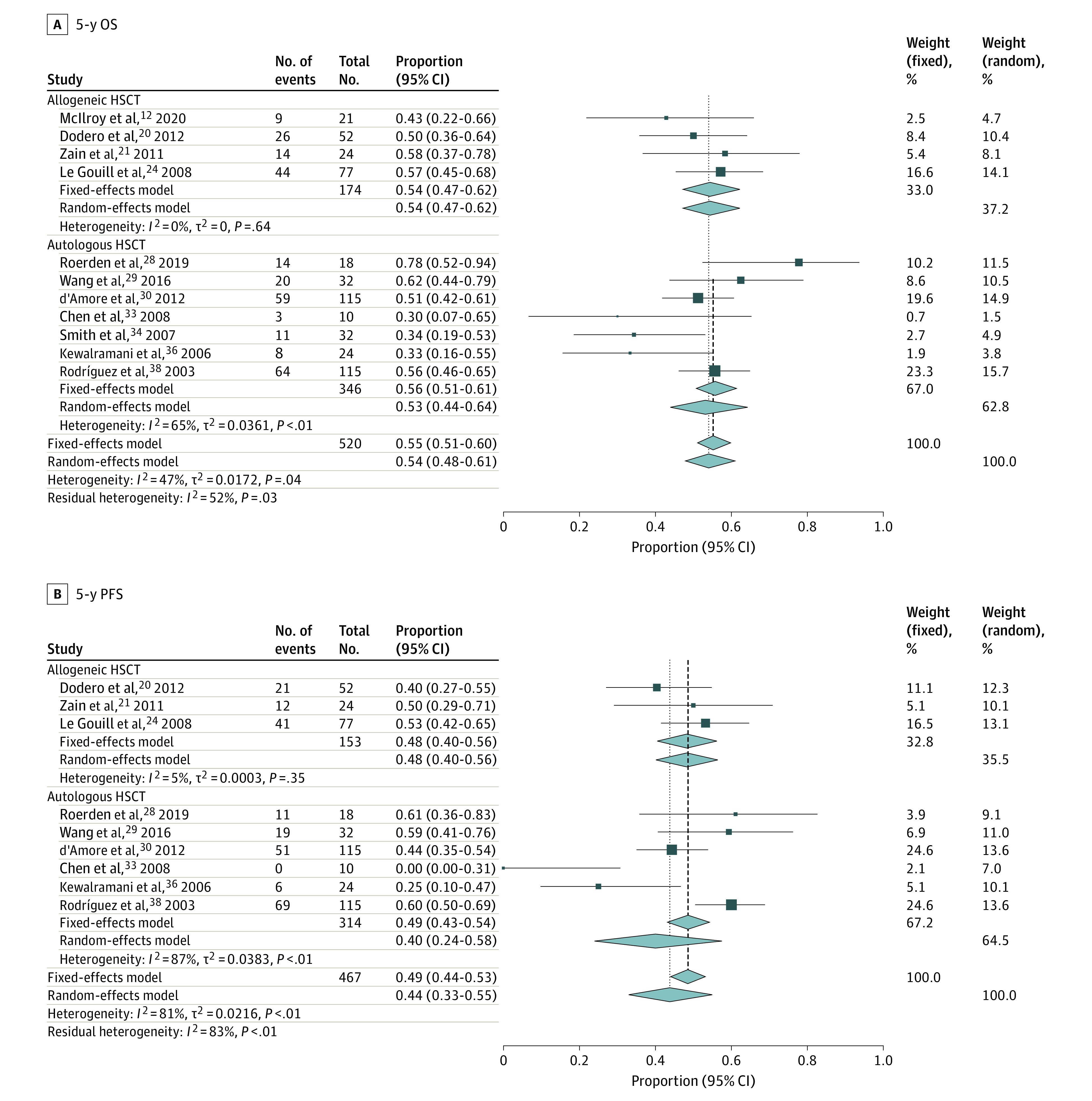
Five-Year Survival Outcomes in Patients with Refractory or Relapsed Peripheral T-Cell Lymphoma HSCT indicates hematopoietic stem cell transplant; OS, overall survival; PFS, progression-free survival.

The outcomes can be classified into 2 categories: survival measurements and AEs. Survival measurements included OS, PFS (including event-free survival and disease-free survival), AEs (including TRM and NRM), and GVHD incidence. Overall survival is the time from receiving transplantation to death from any cause, and PFS is the time from undergoing transplant to relapse or progressive disease or death from any cause. Patients who underwent autologous HSCT would not have a GVHD effect. We performed a subgroup analysis according to different transplant types and different observation times (Figure 2 and Figure 3). Therefore, a total of 6 subgroups were evaluated, including OS at 3 years and 5 years, PFS at 3 years and 5 years, and TRM at 3 years and 5 years.

### Association of Transplant Type With OS

#### OS at 3 Years

Sixteen trials^5,13,14,15,16,18,19,22,23,26,27,30,31,32,35,39^ with 1002 patients reported OS at 3 years. Nine trials^13,14,15,16,18,19,22,23,26^ with 388 patients were assessed for 3-year OS (*I*^2^ = 71%) in the allogeneic HSCT group, and the pooled 3-year OS was 50% (95% CI, 41%-60%) for all patients with R/R-PTCL treated with allogeneic HSCT. The autologous HSCT group included 9 studies^5,16,18,27,30,31,32,35,39^ with 614 patients, and the pooled 3-year OS was 55% (95% CI, 48%-64%). This finding indicates that patients who underwent autologous HSCT might have a relatively better OS than those who underwent allogeneic HSCT after 3 years of follow-up. However, on the basis of the pretransplant CR rate of allogeneic HSCT and autologous HSCT, 42.60% vs 63.37% (χ^2^ = 47.20, *P* < .001), which we calculated via SPSS software, version 25.0 (SPSS Inc), we found that the patients undergoing autologous HSCT were commonly sensitive to chemotherapy or experienced their first CR after induction therapy. Therefore, when we take the patient enrollment bias into consideration, allogeneic HSCT prolonged the 3-year OS overall, especially for those who did not acquire CR before transplantation.

#### OS at 5 Years

Eleven studies^12,20,21,24,28,29,30,33,34,36,38^ with 520 patients provided a 5-year OS for the 2 groups. Allogeneic HSCT had a combined 5-year OS of 54% (95% CI, 47%-62%) across 4 trials^12,20,21,24^ with 174 patients. Autologous HSCT had an integrated 5-year OS of 53% (95% CI, 44%-64%) across 7 trials^28,29,30,33,34,36,38^ with 346 patients. This result suggests that compared with autologous HSCT, the effectiveness of allogeneic HSCT was not different concerning the 5-year OS. Considering that autologous HSCT is a commonly accepted strategy for R/R-PTCL, whereas allogeneic HSCT occasionally serves as a salvage approach, allogeneic HSCT performed better for R/R-PTCL than autologous HSCT. The finding that survival for patients with R/R-PTCL receiving allogeneic HSCT at 3 years was lower than that at 5 years might be because the article sources are distinct, so OS could not be compared directly, and the patient baseline characteristics should be taken into consideration.

### DFS, EFS, and PFS

Not all studies presented PFS, EFS, and DFS; therefore, we combined these variables and unified them as PFS to reveal the data more clearly. In the 11 trials^13,14,15,16,18,20,21,22,23,24,26^ that reported the PFS end point for the allogeneic HSCT group, the combined PFS was 42% (95% CI, 35%-51%) at 3 years (8 trials^13,14,15,16,18,22,23,26^) and 48% (95% CI, 40%-56%) at 5 years (3 trials^20,21,24^). In the 15 studies^5,16,18,27,28,29,30,31,32,33,35,36,38,39^ that reported the PFS end point for the autologous HSCT group, the combined PFS was 41% (95% CI, 33%-51%) at 3 years (9 trials^5,16,18,27,30,31,32,35,39^) and 40% (95% CI, 24%-58%) at 5 years (6 trials^28,29,30,33,36,38^). Confounding bias still existed in the 5-year outcome in the allogeneic HSCT group; therefore, we could compare the therapy efficiency based only on 3-year PFS, which was 42% (95% CI, 35%-51%) for the allogeneic HSCT group and 41% (95% CI, 33%-51%) for the autologous HSCT group, indicating they were approximately equivalent to each other in terms of the PFS at 3 years.

### TRM

As with the PFS, EFS, and DFS, we performed statistical analysis of TRM by combining the data of TRM and NRM and labeled them together as TRM. Six trials reported a pooled 3-year TRM of 32% (95% CI, 27%-37%) in the allogeneic HSCT group, and 3 trials reported a pooled 3-year TRM of 7% (95% CI, 2%-23%) in the autologous HSCT group, suggesting higher TRM with allogeneic HSCT. The TRM at 5 years for R/R-PTCL patients was 24% (95% CI, 6%-95%) in the allogeneic HSCT group and 55% (95% CI, 32%-97%) in the autologous HSCT group.

## Discussion

This systematic review and meta-analysis reviewed studies performed from 2001 to 2020 on transplant for R/R-PTCL, reporting outcomes of allogeneic HSCT and autologous HSCT for R/R-PTCL. Patients in the 2 groups had similar survival rates, whereas patients with R/R-PTCL who underwent autologous HSCT had fewer AEs than who underwent allogeneic HSCT, likely because GVHD counterbalances the accompanying graft-vs-lymphoma effect after allogeneic HSCT.^40^ However, considering the pretransplant status, most patients in the allogeneic HSCT group were insensitive to chemotherapy, and allogeneic HSCT served as a salvage therapy, which provided an additional survival advantage for patients with R/R-PTCL. These findings might be linked to the graft-vs-lymphoma effect.

Peripheral T-cell lymphoma is rare, accounting for a small proportion of all non-Hodgkin lymphoma cases (6%-10%), with approximately 4800 to 8000 new cases per year in the US. Coupled with the diversified histologic findings (29 subtypes), personalized precision therapy is hard to establish. Especially in the R/R-PTCL setting, no ideal therapies have been developed, resulting in a dismal prognosis with high expenditure.^6^ The poor prognosis of patients with R/R-PTCL has always been concerning. Therefore, efficient treatment strategies should be explored for patients with R/R-PTCL. In the past 20 years, with the development of medical technology and the improvement of economic conditions, more patients, especially those in developing countries, have the opportunity to receive HSCT, which is the mainstay treatment for patients with PTCL. High-density chemotherapy combined with autologous HSCT has become a conventional treatment method for patients with PTCL at first CR. However, for R/R-PTCL, the preferred option between allogeneic HSCT and autologous HSCT is still controversial.

Compared with allogeneic HSCT, more patients are eligible for autologous HSCT with less expenditure. Furthermore, no GVHD occurred in the autologous HSCT group. However, stem cells transplanted to patients may have a higher possibility of containing tumor cells, and no obvious graft-vs-lymphoma effect occurred in the autologous HSCT group, leading to an increased incidence of relapse compared with the allogeneic HSCT group. However, the outcome may be different for few specific histologic subtypes. The European Cooperative Group for Bone Marrow Transplantation demonstrated that favorable outcomes are seen in patients with angioimmunoblastic T-cell lymphoma angioimmunoblastic T-cell lymphoma undergoing allogeneic HSCT.^41^ In the current study, patients undergoing autologous HSCT had a 3-year OS of 55% and PFS of 41% and a 5-year OS of 53% and PFS of 40%, showing a similar OS for patients undergoing autologous HSCT compared with those undergoing allogeneic HSCT.

Some studies^16,18,42^ compared OS between allogeneic HSCT and autologous HSCT in patients with PTCL. A large retrospective trial^16^ that included 67 patients found a 3-year OS of 53%, a 3-year PFS of 49%, and a 1-year NRM of 18% for patients undergoing allogeneic HSCT (n = 24) and a 3-year OS of 20%, a 3-year PFS of 20%, and a 1-year NRM of 7% for patients undergoing autologous HSCT (n = 43),^16^ indicating that favorable survival outcomes were observed in the allogeneic HSCT group. Nevertheless, Smith et al^18^ reported allogeneic HSCT outcomes in 126 patients with R/R-PTCL, with a 3-year OS of 46%, 3-year PFS of 37%, and 3-year NRM of 34%. In addition, 115 patients with R/R-PTCL undergoing autologous HSCT had a 3-year OS of 59%, 3-year PFS of 47%, and 3-year NRM of 6%.^18^ These 2 trials^16,18^ were included in the current study. To identify whether they would affect the conclusions, a sensitivity analysis was performed. After these 2 studies^16,18^ were omitted, similar results were found (eAppendix in the Supplement). Moreover, the AATT study^42^ with 103 patients reported no significant difference between allogeneic HSCT and autologous HSCT as first-line therapy, with a 3-year OS of 57% in allogeneic HSCT and 70% in autologous HSCT.^42^ Furthermore, a systematic review^43^ of allogeneic HSCT and autologous HSCT published in 2015 found no difference in OS. However, O’Connor et al^44^ noted that because of the different disease stages before transplantation between the 2 groups of patients, allogeneic HSCT provides extra survival advantages for patients with R/R-PTCL.^6^

As demonstrated in this study, OS is not significantly different between different HSCT types; therefore, cutting-edge treatment strategies need to be explored. For example, using specific HSCT types for patients with PTCL with different risk stages is a recommended strategy. Autologous HSCT was used as the first-line therapy, and allogeneic HSCT was used when patients had R/R-PTCL. However, HSCT should not be recommended later than second-line therapy if patients who are ready for transplant have already undergone multiline treatment because the prognosis will be worse.^11^ Novel treatments, such as applying chimeric antigen receptor T or natural killer cells or developing multidrug combinations, can also be tried. Combinational medicine (particularly epigenetic drugs) was also suggested to improve the curative effect for patients with PTCL, especially for angioimmunoblastic T-cell lymphoma.^45^

Researching the biological heterogeneity of T cells is of vital significance to reform the treatment strategies for PTCL, and multicenter randomized clinical trials should be conducted. Because of the paucity of patient numbers, case-matched control studies can be based on historical comparative trials,^44^ which is a reasonable analysis method for this orphan disease. In the future, developing targeted therapy and combining existing drugs as well as optimizing the transplant system are crucial goals.

### Limitations

This study has limitations and biases, even though strict enrollment criteria were set. First, most of the eligible studies for inclusion were single-arm trials, so results could not be directly evaluated. Second, the data for some outcome measurements were too scarce to perform a subgroup analysis, resulting in heterogeneity. Third, there was wide variation in the included patients with R/R-PTCL, such as age, histologic findings, and treatment characteristics, which would have an impact. Although this study did not fulfill the above features completely, overall the bias risk of study quality was acceptable.

## Conclusions

A historical retrospective conclusion about the ideal means to treat R/R-PTCL for 20 years was drawn from this meta-analysis. Patients with R/R-PTCL undergoing allogeneic HSCT and autologous HSCT had similar survival conditions, whereas GVHD and higher TRM occurred in the allogeneic HSCT group. However, because of the CR status before transplant, allogeneic HSCT was associated with a specific survival advantage over autologous HSCT. The findings of this study suggest that, overall, HSCT is an effective therapy for R/R-PTCL. Patients with R/R-PTCL with lower-risk stratification might prefer autologous HSCT, although allogeneic HSCT still serves as the cornerstone of salvage therapy in those with a higher-risk disease stage. In the future, multicenter collaboration should be performed to optimize treatment for patients with R/R-PTCL patients.^46^

## Notes

Supplement. **eFigure 1.** MINORS Scale **eFigure 2.** Bubble Chart Describing the Years and Sample Size **eFigure 3.** Heatmap of MINORS Scale to Assess the Study Quality **eFigure 4.** Four Funnel Plots to Estimate Publication Bias **eFigure 5.** Forest Plots of Survival Outcomes After Omitting Two Comparative Studies
