# Supplementary material for: Comparison of Allogeneic Stem Cell Transplant and Autologous Stem Cell Transplant in Refractory or Relapsed Peripheral T-Cell Lymphoma: A Systematic Review and Meta-analysis
Source: JAMA Netw Open. 2021 May 27;4(5):e219807. doi: 10.1001/jamanetworkopen.2021.9807 (PMC8160596; doi:10.1001/jamanetworkopen.2021.9807)
Supplement: Supplement. — eFigure 1. MINORS Scale eFigure 2. Bubble Chart Describing the Years and Sample Size eFigure 3. Heatmap of MINORS Scale to Assess the Study Quality eFigure 4. Four Funnel Plots to Estimate Publication Bias eFigure 5. Forest Plots of Survival Outcomes After Omitting Two Comparative Studies [file jamanetwopen-e219807-s001.pdf]

Supplementary Online Content

Du J, Yu D, Han X, Zhu L, Huang Z. Comparison of allogenic stem cell transplant and autologous stem cell transplant in refractory or relapsed peripheral T-cell lymphoma: a systematic review and meta-analysis. *JAMA Netw Open*. 2021;4(5):e219807. doi:10.1001/jamanetworkopen.2021.9807

eFigure 1. MINORS Scale

eFigure 2. Bubble Chart Describing the Years and Sample Size

eFigure 3. Heatmap of MINORS Scale to Assess the Study Quality

eFigure 4. Four Funnel Plots to Estimate Publication Bias

eFigure 5. Forest Plots of Survival Outcomes After Omitting Two Comparative Studies

This supplementary material has been provided by the authors to give readers additional information about their work.

© 2021 Du J et al. *JAMA Network Open.*

eFigure 1. MINORS Scale

Methodological items for non-randomized studies	Score†
1. **A clearly stated aim:** the question addressed should be precise and relevant in the light of available literature<br>2. **Inclusion of consecutive patients**: all patients potentially fit for inclusion (satisfying the criteria for inclusion) have been included in the study during the study period (no exclusion or details about the reasons for exclusion)<br>3. **Prospective collection of data**: data were collected according to a protocol established before the beginning of the study<br>4. **Endpoints appropriate to the aim of the study**: unambiguous explanation of the criteria used to evaluate the main outcome which should be in accordance with the question addressed by the study. Also, the endpoints should be assessed on an intention-to-treat basis.<br>5. **Unbiased assessment of the study endpoint**: blind evaluation of objective endpoints and double-blind evaluation of subjective endpoints. Otherwise the reasons for not blinding should be stated<br>6. **Follow-up period appropriate to the aim of the study**: the follow-up should be sufficiently long to allow the assessment of the main endpoint and possible adverse events<br>7. **Loss to follow up less than 5%**: all patients should be included in the follow up. Otherwise, the proportion lost to follow up should not exceed the proportion experiencing the major endpoint<br>8. **Prospective calculation of the study size**: information of the size of detectable difference of interest with a calculation of 95% confidence interval, according to the expected incidence of the outcome event, and information about the level for statistical significance and estimates of power when comparing the outcomes	
Additional criteria in the case of comparative study<br>9. **An adequate control group**: having a gold standard diagnostic test or therapeutic intervention recognized as the optimal intervention according to the available published data<br>10. **Contemporary groups**: control and studied group should be managed during the same time period (no historical comparison)<br>11. **Baseline equivalence of groups**: the groups should be similar regarding the criteria other than the studied endpoints. Absence of confounding factors that could bias the interpretation of the results<br>12. **Adequate statistical analyses**: whether the statistics were in accordance with the type of study with calculation of confidence intervals or relative risk	

†The items are scored 0 (not reported), 1 (reported but inadequate) or 2 (reported and adequate). The global ideal score being 16 for non-comparative studies and 24 for comparative studies.

*eFigure 1 is a screenshot took from the reference (Slim K, et al[9].)

© 2021 Du J et al. *JAMA Network Open.*

eFigure 2. Bubble Chart Describing the Years and Sample Size

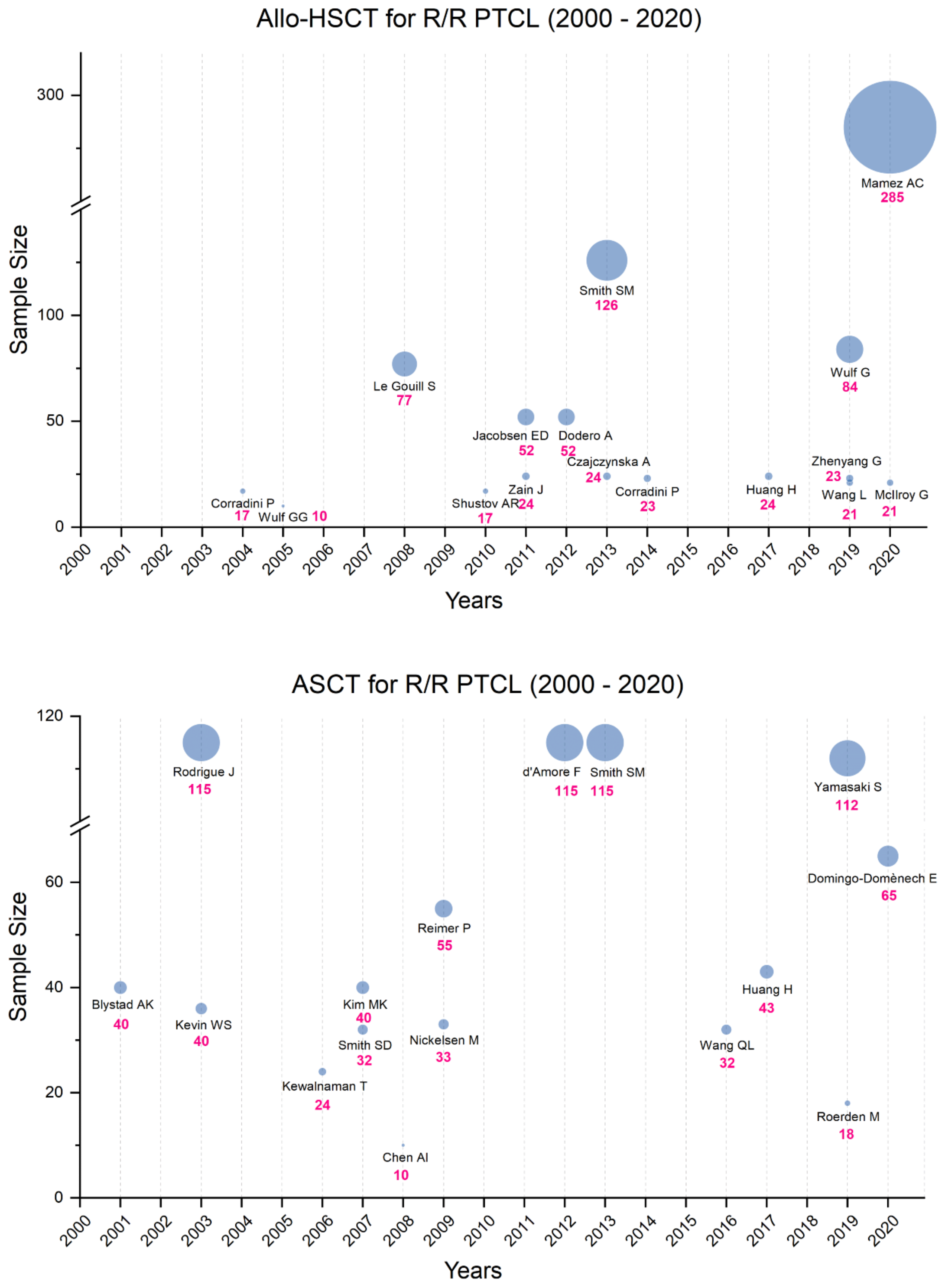

© 2021 Du J et al. *JAMA Network Open.*

eFigure 3. Heatmap of MINORS Scale to Assess the Study Quality

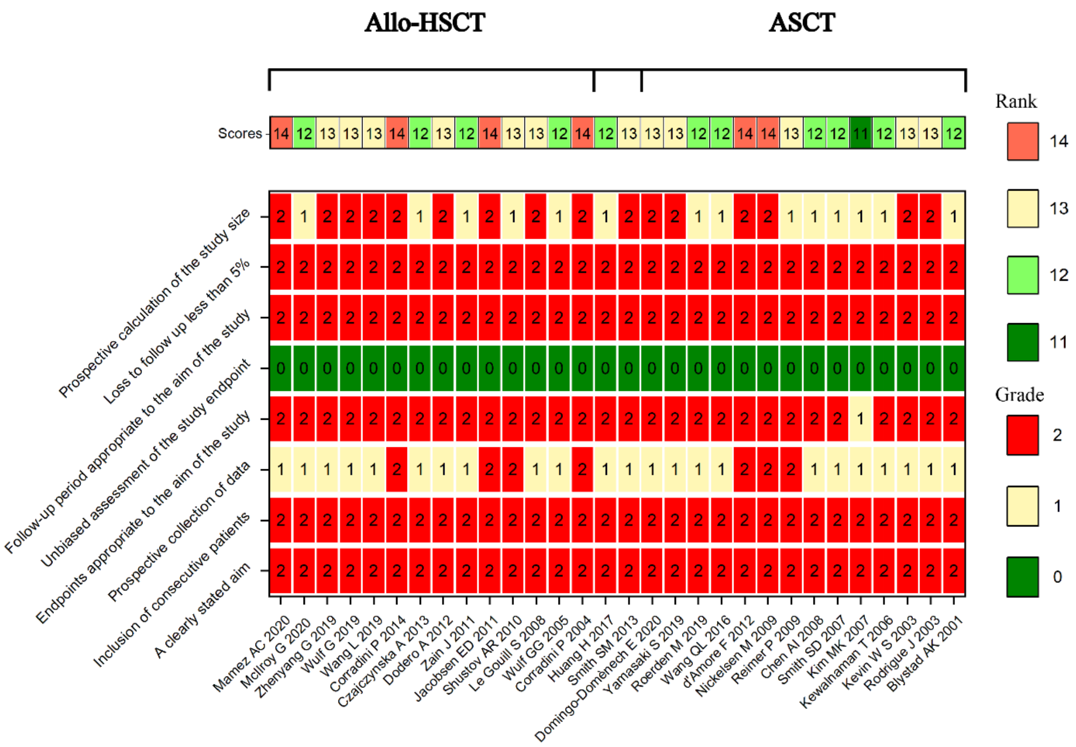

The items are scored 0 (not reported), 1 (reported but inadequate) or 2 (reported and adequate).

© 2021 Du J et al. *JAMA Network Open.*

eFigure 4. Four Funnel Plots to Estimate Publication Bias

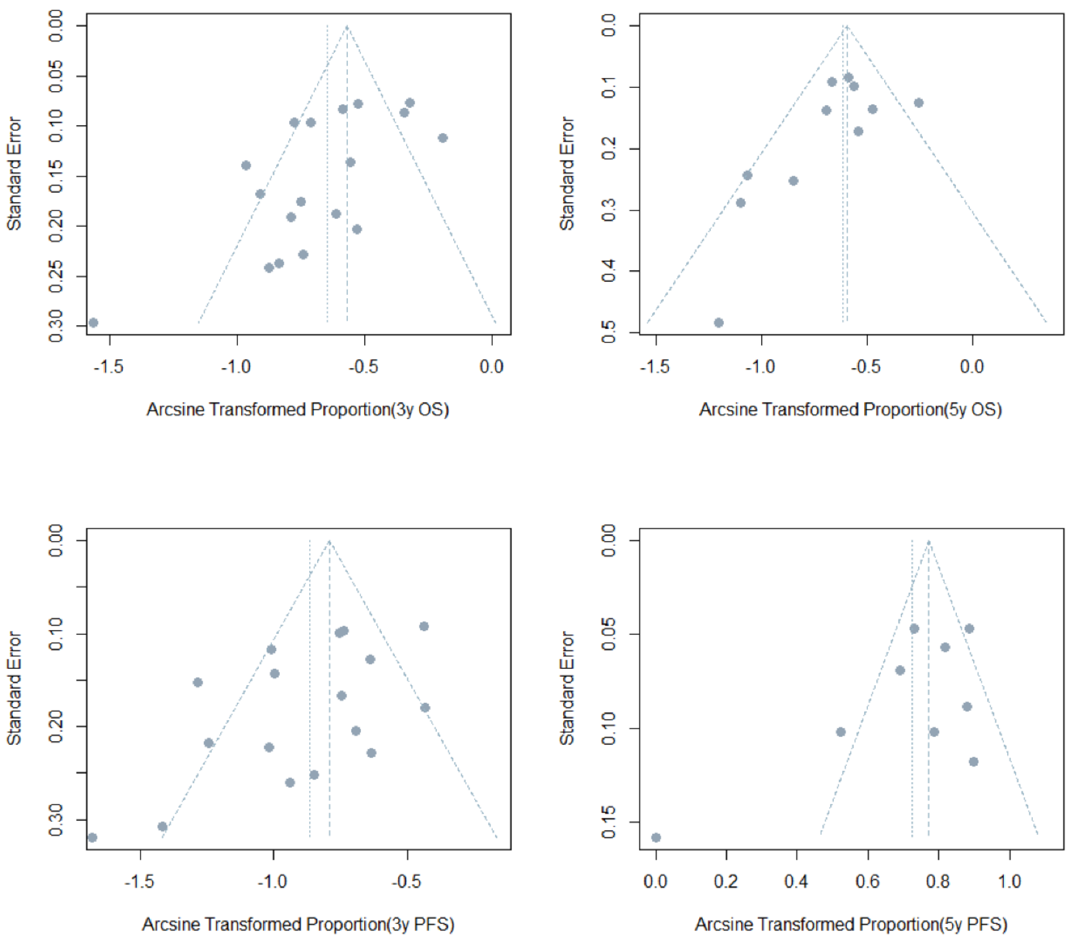

© 2021 Du J et al. *JAMA Network Open.*

eFigure 5. Forest Plots of Survival Outcomes After Omitting Two Comparative Studies

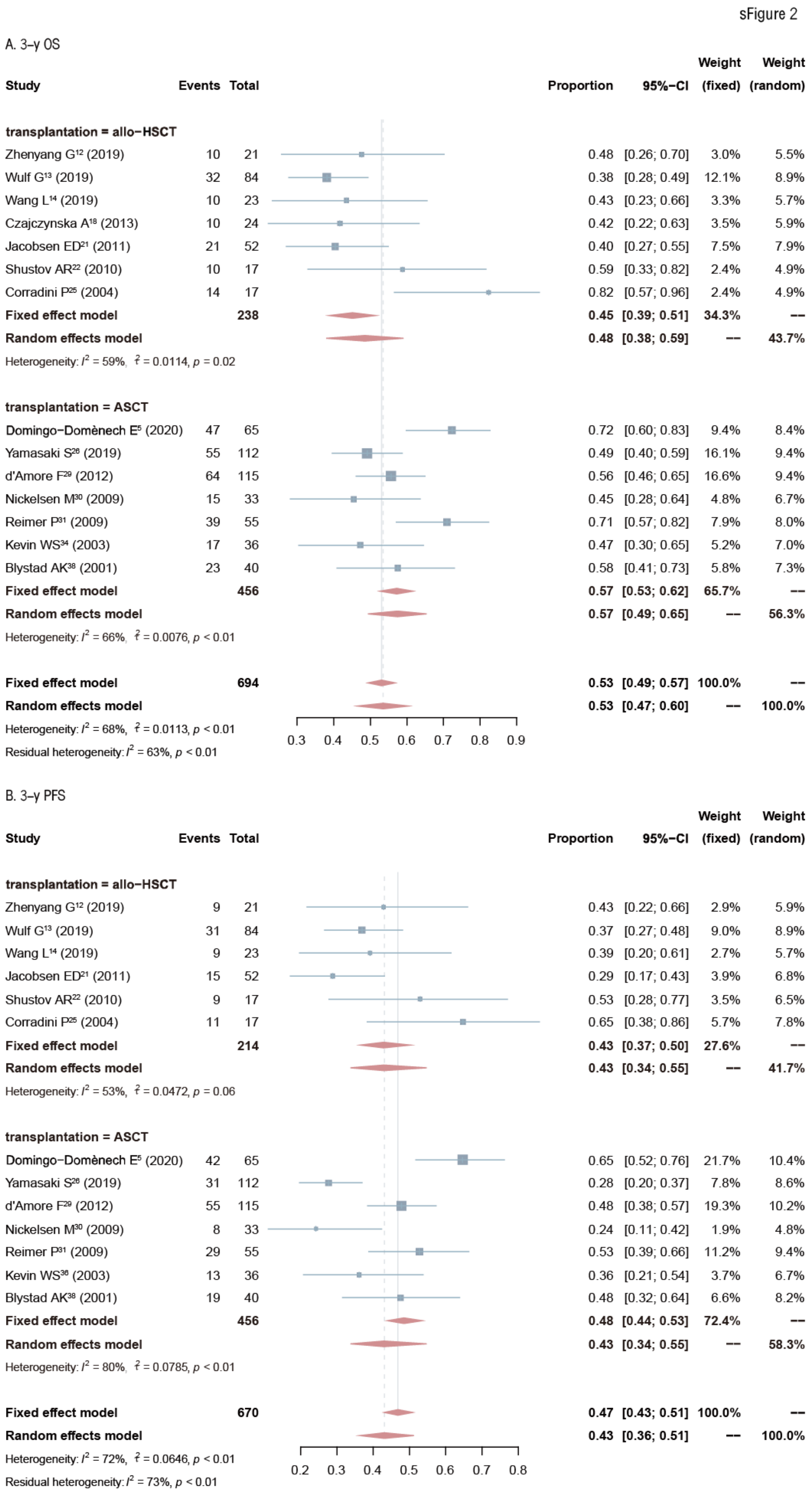

© 2021 Du J et al. *JAMA Network Open.*

TRM/NRM

Similar to PFS/EFS/DFS, we performed statistical analysis by combining the data of TRM and NRM and labeled them together as TRM. As a whole, the number of studies that denoted the TRM at 3 years or 5 years was relatively small, especially at 5 years, with only two studies for each type. Six trials reported a pooled 3-year TRM of 32%(95%CI, 27-37%) in the allo-HSCT group, and three trials reported a pooled 3-year TRM of 7%(95%CI, 2-23%) in the ASCT group, showing a trend toward higher TRM with allo-HSCT than with ASCT. The TRM at 5 years for R/R-PTCL patients was 24%(95%CI, 6-95%) in the allo-HSCT group and 55%(95%CI, 32-97%) in the ASCT group. We can omit the 5-year TRM because those statistics were nonsensical. In summary, ASCT seems to be a more advisable treatment option given the lower possibility of R/R-PTCL patients suffering transplantation-related mortality. For allo-HSCT, TRM is still a difficult challenge to address in order to make a break-through. Thus, improving the preconditioning regimen of transplantation and promoting the comprehensive prophylaxis therapy of GVHD is a recommended strategy.

GVHD

In the allo-HSCT group, the incidence of grade II-IV acute GVHD (aGVHD) ranged from 14 to 40%, that of limited chronic GVHD (cGVHD) ranged from 5 to 50%, and that of extensive cGVHD ranged from 6 to 54%. No significant trend was observed regarding the GVHD incidence over time. As shown in the article with the largest sample size (n=285), Mamez AC and Dupont A reported that 30%of the patients had grade II–IV acute GVHD (grade III–IV = 14.7%), and chronic GVHD occurred in one-third of the patients (extensive in 14.8%).

© 2021 Du J et al. *JAMA Network Open.*
